# Retraction Note: therapeutic stimulation of GLP-1 and GIP protein with DPP-4 inhibitors for type-2 diabetes treatment

**DOI:** 10.1186/s40200-016-0256-4

**Published:** 2016-08-30

**Authors:** Alok Sharma, Geetanjali Paliwal, Nisha Upadhyay, Archana Tiwari

**Affiliations:** School of Biotechnology, Rajiv Gandhi Proudyogiki Vishwavidyalaya (State Technological University of Madhya Pradesh), Bhopal, India

The Editors are retracting this article [1] due to overlap with previous publications, most notably [2, 3]. We apologise for any inconvenience caused. We have been unable to contact the authors regarding the retraction of their article.
